# Investigation of bioimpedance as a method for wearable noninvasive bladder volume measurements in individuals with spinal cord injury or disease: protocol of a feasibility study

**DOI:** 10.3389/fruro.2026.1799957

**Published:** 2026-06-11

**Authors:** Judith Jantine Willemijn van Beek, Kanika Dheman, Sabrina Amrein, Michele Magno, Diego Paez-Granados, Jürgen Pannek, Jörg Krebs

**Affiliations:** 1 Neuro-Urology, Swiss Paraplegic Research, Nottwil, Switzerland; 2 D-ITET Center for Project-Based Learning, ETH Zurich, Zurich, Switzerland; 3 SCAI lab, Department of Health Sciences and Technology, ETH Zurich, Zurich, Switzerland; 4 Digital Health Care and Rehabilitation, Swiss Paraplegic Research, Nottwil, Switzerland; 5 Neuro-Urology, Swiss Paraplegic Centre, Nottwil, Switzerland; 6 Department of Urology, Inselspital, Bern University Hospital, University of Bern, Bern, Switzerland; 7 Medicine and Medical Sciences, Health Sciences and Medicine, University of, Lucerne, Switzerland

**Keywords:** bioimpedance sensor, bladder volume monitoring, neurogenic lower urinary tract dysfunction, noninvasive, spinal cord injury/disease

## Abstract

**Introduction:**

Neurogenic lower urinary tract dysfunction (NLUTD) is a frequent consequence of spinal cord injury or disease (SCI/D). It can lead to impaired bladder emptying, urinary incontinence, and recurrent urinary tract infections. For those unable to empty their bladder effectively, intermittent catheterization (IC) is the recommended first-line management. Clinical guidelines advise catheterization four to six times daily to prevent bladder overdistension (i.e., bladder volume > 500 ml), yet fixed schedules are often impractical due to fluctuating daily factors. Personalized scheduling is particularly challenging for individuals with SCI/D who lack bladder sensation. Consequently, a noninvasive and unobtrusive method for bladder volume monitoring is needed. Electrical bioimpedance (BI) is a promising solution, as it measures the impedimetric response of tissue to an applied electrical signal and should detect changes related to bladder filling. This may enable continuous, dynamic volume estimation and support individualized bladder management.

**Methods and analysis:**

This multi-center observational feasibility study evaluates a BI sensor for noninvasive bladder volume monitoring across three phases. Phase 1 assesses feasibility in individuals without SCI/D. Phase 2 includes individuals with chronic SCI/D and NLUTD to determine the difference between BI-derived bladder volume and the clinical gold standard (bladder volume determined by catheterization). Effects of abdominal adiposity and muscle activity on BI-signals will also be analyzed. Phase 3 examines the usability of a BI-based wearable during six hours of real-life use in wheelchair users reliant on intermittent catheterization. Sixty participants will be enrolled across all phases. The primary outcome is the difference in bladder volume (mL) between BI measurements and catheterized volumes in individuals with SCI/D. Secondary outcomes include physiological and motion parameters possibly affecting BI measurements. Analyses will be descriptive, focusing on signal quality, feasibility, and measurement agreement.

**Discussion:**

This multi-phase feasibility study evaluates whether a BI sensor can measure bladder volume in individuals with SCI/D and NLUTD noninvasively and continuously. By validating BI in controlled and real-life settings, the study aims to determine its feasibility and potential as an alternative to current bladder volume assessment methods.

## 1 Introduction

Neurogenic lower urinary tract dysfunction (NLUTD) is a common consequence of spinal cord injury/disease (SCI/D), affecting approximately 70-84% of individuals living with SCI/D (1). A variety of complications, including impaired bladder emptying, urinary incontinence, recurrent urinary tract infections (UTI), and renal damage can result from NLUTD (2). The recommended first-line treatment for individuals unable to empty their bladder effectively is intermittent catheterization (IC) (2, 3). Guidelines from the European Association of Urology (3, 4), and the American Urological Association (5, 6), recommend IC four to six times per day to avoid bladder overdistension (≥ 500 ml per catheterization). However, adherence to a fixed schedule is often impractical due to varying factors such as food and fluid intake, activities and daily life participation. Thus, IC schedules must be tailored according to individual needs and bladder function, which is especially challenging for people with SCI/D lacking bladder sensation. Poorly managed bladder function can lead to urinary incontinence, which causes stress and reluctance to engage in social activities (2). In persons with SCI/D above T6, it can lead to autonomic dysreflexia, a potentially life-threatening complication. Therefore, there is a need for a noninvasive, unobstructive method to continuously monitor bladder volume to optimize catheterization timing and prevent complications associated with bladder overfilling.

A promising noninvasive technology for bladder volume monitoring is the use of electrical bioimpedance (BI) (7). BI measures the impedimetric response of biological matter to an externally applied electrical signal (8), allowing estimation of bladder volume over time by detecting impedance changes during changes in intravesical bladder volume. This continuous measurement enables dynamic monitoring rather than intermittent snapshots, which is key to personalized bladder management. Additionally, BI devices offer advantages including ease of use and improved patient comfort, as they are noninvasive, portable, and can be integrated into wearables (7). The BI method involves applying a small high-frequency electric current to a specific body segment (e.g., the lower abdomen), and measuring the reflected voltage changes by that segment using surface electrodes (7, 9–11). BI has been studied extensively for noninvasive, safe and inexpensive assessment of body composition in both clinical and home settings (12, 13). In particular, the method has been studied for scenarios where the body’s fluid composition changes over time, such as during dehydration and edema (14).

This multi-center observational study aims to evaluate the feasibility of the BI sensor to reliably measure bladder volume. The objective of this study is not the clinical validation of a finalized bladder monitoring device, but rather the systematic investigation of feasibility, sensitivity, and practical limitations of wearable bioimpedance measurements for monitoring intravesical volume changes across individuals with diverse physiologies and real-world usage conditions. Thus, we will assess the initial prototype performance and optimize the device across three phases. In phase 1, we will evaluate the feasibility of the BI sensor in individuals without SCI/D and NLUTD. Phase 2 will focus on individuals with SCI/D and NLUTD, including analysis of the impact of abdominal adipose tissue on BI measurement sensitivity by comparing BI-estimated bladder volume with the gold standard for bladder volume measurement, namely catheterization (3). In phase 3, we will evaluate the usability of a BI-based wearable in the SCI/D population during six hours of real-life use.

## 2 Methods and analysis

### 2.1 Study design

This is a multi-center observational feasibility study. The study comprises three phases conducted at two locations in Switzerland. Ethical approval has been given by the competent ethics committees. The study adheres to the Standardized Protocol Items Recommendations for Observational Studies (SPIROS) guideline (SPIROS 2024 - Responsible Reporting - SPIROS 2024 - Responsible Reporting - Maastricht University).

### 2.2 Study setting

Phase 1 will take place at a technical university where employees and students at the university without SCI/D will be recruited via email to or in-person contact. Phases 2 and 3 will take place in a specialized center for acute care and rehabilitation of persons with SCI/D. For phase 2, the clinical planning system will be screened for potential study participants and eligible individuals will be approached at least 24 hours prior to their next scheduled video-urodynamic studies (VUDS) in the department of neuro-urology. For phase 3, peer counsellors and employees with SCI/D will be recruited.

### 2.3 Sample size

In line with feasibility study conventions, no formal sample size calculation was performed; each study phase will recruit a minimum of 10 participants, which is considered adequate to assess methodological feasibility and to guide future sample size estimations (15).

### 2.4 Participant selection

A total of 60 participants will be recruited across all phases: 20 in phase 1, 30 in phase 2, and 10 in phase 3. The larger sample in phase 2 reflects the greater heterogeneity of the SCI/D population and the need to evaluate performance across different injury characteristics and bladder management strategies. Participant dropouts will be replaced until the target sample size is reached. All participants must be at least 18 years old.

Inclusion criteria per phase:

- Phase 1: Individuals (10 female and 10 male) without SCI/D.
- Phase 2: Individuals with chronic SCI/D (≥12 months) and NLUTD, scheduled for VUDS.
- Phase 3: Wheelchair users with SCI/D (≥3 months) who rely on IC for bladder evacuation.

Exclusion criteria for all phases include dependent relationship with members of the study team, existing skin disorders, any implants in the abdominal area, presence of suprapubic or transurethral catheters, and any morphological or physiological conditions that prevent proper sensor attachment (e.g., spasticity, extensive scarring or malformations).

### 2.5 Procedures

The study objectives, procedures, potential risks and benefits will be explained to all potential participants. Written informed consent will be obtained prior to inclusion into the study in accordance with national regulations and international guidelines (16). Participation is voluntary, and participants may withdraw at any time without justification.

#### 2.5.1 Phase 1: measurements in individuals without SCI/D

Bladder volume will be measured in 20 participants during spontaneous voiding, with uroflowmetry serving as the reference standard.

- One hour before the examination, participants will drink 500 mL of water.
- Anthropometric data will be recorded.
- The BI sensor consisting of four electrodes and an electromyography (EMG) channel will be placed on the lower abdomen as described by Dheman et al. (17, 18).
- BI-derived bladder volume changes and abdominal muscle activity will be recorded. Participants will empty their bladders when they feel the urge to void. They will then void into a special funnel connected to an electronic uroflowmeter, which measures both the urine output (volume) and flow rate.
- After voiding, half of the participants (five female, five male) will complete an additional standardized activities of daily living (ADL) protocol designed for wheelchair users. This protocol is used to assess susceptibility to motion artifacts. A study member will observe and document how long each activity takes. The activity protocol lasts about 20 minutes and includes active and passive propulsion, simple arm raises and transferring between a wheelchair and a chair/sofa.

The total duration of the examination for each participant will be 45–60 minutes.

#### 2.5.2 Phase 2: measurements in individuals with SCI/D undergoing routine VUDS

Prior to the VUDS, participants will be equipped with the BI sensor as described in phase 1. They will also be equipped with an electrocardiography (ECG) patch (VivaLNK C, USA) placed on the left side of the chest. This device records various physiological parameters, including ECG rhythm, heart rate, heart rate variability, R-R interval, respiratory rate, skin temperature, and 3-axis accelerometer data (19). In addition, participants will get a photoplethysmography wristband (Cardiowatch Bracelet 287-2, Corsano Health TH, The Netherlands) on the right wrist. This device measures wrist acceleration, skin temperature, heart rate and respiratory rate intervals (20).

After sensor placement, participants will undergo VUDS according to the International Continence Society standards (21). During the routine clinical procedures, bladder volume will be assessed using BI. At the end of the examination, the total bladder volume will be measured via catheterization. The complete examination will last 45–105 minutes, including 30–90 minutes required for the standard clinical procedures.

#### 2.5.3 Phase 3: continuous measurements in individuals with SCI/D during daily activities

Participants will be equipped with the BI sensor, ECG patch and photoplethysmography wristband as described in the previous phases. In addition, the participants’ wheelchair will be equipped with a pressure mat (sensomative, Switzerland) placed under the regular cushion to measure pressure distribution (22), and an acceleration sensor (MMR-MMC Mbient) attached on the right wheel axle (MbientLab Inc SF, USA) (23). All five sensors will be worn continuously for 6 hours. Participants will also receive a smartphone that collects sensor data.

A calibration bladder void will be performed by the participants, and the voided urine volume measured using a calibrated container. Participants will then complete the same standardized ADL protocol described in phase 1. This sequence will be recorded on video. Thereafter, measurements will continue during normal ADL without video recording. After the 6-hour monitoring period, a study member will retrieve all sensors.

### 2.6 BI sensor

The existing prototype of the sensor node, which measures BI of the lower abdomen, has previously been tested in six healthy adults (17). The prototype consists of a battery-operated embedded device with both wireless processing unit and sensing interface, the device casing, and four electrode attachment sites. It acquires measurements at 300 ms intervals and stores the data on a secure digital card.

The sensor node is not directly attached or in contact with the participant’s body, but connected via CE certified electrodes (AMBU^®^, AMBU A/S, Denmark), minimizing potential risks. The system was designed, manufactured, and tested according to medical device development standards and regulations (ISO 14971:2018, IEC60601-1:2005). The average measurement error was comparable to that of commercial handheld ultrasound devices. The system operates on a low power certified 3.7 V LiPo battery, which does not come into direct contact with participants. During use, the device is placed on a nearby table or in a wheelchair pouch, preventing the risk of battery-related skin irritation.

### 2.7 Outcome measures

#### 2.7.1 Primary outcome

The primary outcome is the difference in bladder volume (mL) between BI measurements and bladder volume measured by catheterization in individuals with SCI/D.

#### 2.7.2 Secondary outcomes

Secondary outcomes are selected to (1) support validation of BI-based bladder volume estimations, (2) quantify physiological and technical confounders known to influence impedance signals, and (3) evaluate feasibility of the sensor under controlled and real-world conditions.

Phase 1:

- spontaneously voided urine volume
- urine flow rate

These outcomes provide reference information on bladder emptying dynamics and voided volume to support initial validation of BI-derived volume changes under controlled physiological conditions.

Phase 2:

- VUDS parameters (i.e., bladder volume, maximal storage detrusor pressure, detrusor compliance, presence of detrusor overactivity)
- total voided urine volume after VUDS

These outcomes serve as clinical reference standards for bladder volume and storage function.

- muscular activity measured by EMG
- ECG data
- heart rate
- respiratory rate
- skin temperature

Physiological outcomes are measured to quantify possible artifacts and autonomic activity that may affect BI measurements.

Phase 3:

- ECG data
- heart rate
- respiratory rate
- skin temperature
- urine volume measured by participants during regular bladder voiding
- 3-axis accelerometer data from integrated inertial measurement units to identify motion artifacts, posture changes, and activity onset (e.g., voiding)
- sitting pressure distribution

These outcomes are used to evaluate signal stability and to enable comparison between BI estimates and real-world voided volumes.

#### 2.7.3 Other outcomes

Other outcomes will include demographic characteristics (i.e. age, sex), clinical characteristics (i.e. etiology, date of injury, level of injury, American Spinal Injury Association Impairment Scale, severity, primary bladder evacuation), and anthropometric data (i.e. weight, height, seated lower abdominal circumference at the level approximately 1 cm below the umbilicus, scapular skin fold, hip-to-shoulder length).

### 2.8 Data analysis

As this is a feasibility study, analyses will be primarily descriptive. Outcomes will be reported as point estimates and 95% confidence intervals (CI). Missing data will not be imputed. Signal completeness, dropout rates, and noise levels will be reported. A *p* value of ≤0.05 (two‐tailed) will be considered significant.

#### 2.8.1 Phase 1 analyses

Bladder volume: The agreement between BI sensor measurements and uroflowmetry will be assessed using Bland-Altman analysis. We will use intraclass correlation coefficient (ICC) (two-way mixed-effects, absolute agreement model) to show how closely the measurements resemble each other overall.

Impact of ADL: BI sensor measurements obtained during the standardized ADL protocol will be analyzed to assess susceptibility to motion artifacts. For each segment, the variability of BI measurements (e.g. standard deviation or coefficient of variation) will be compared between movement and no-movement periods. Signal-to-noise ratios and artifact incidence will be quantified to assess real-world feasibility.

#### 2.8.2 Phase 2 analyses

Bladder volume: The agreement between BI sensor and VUDS will be assessed using Bland-Altman analysis. ICC will be examined to judge the absolute agreement.

Physiological parameters: Changes in heart rate, respiratory rate, skin temperature will be analyzed across predefined bladder-filling and emptying conditions (e.g., pre-filling, 100 mL, 300 mL, fully filled, and post-catheterization) using repeated-measures analysis.

Concurrent EMG will be used to identify potential interference from muscle activity. Additional stratified analyses will evaluate the influence of autonomic dysregulation on BI accuracy.

Associations between the bladder volume differences (primary outcome) and participant characteristics (e.g., abdominal adiposity, sex-related bladder shape differences, anthropometric measures) will be examined using general least squares regression models. In addition to testing the influence of covariates on measurement differences, the models will allow an investigation of the relation between covariates and changes in residual variance (expressing measurement precision).

#### 2.8.3 Phase 3 analyses

Bladder volume: The agreement between BI sensor and container-measured volume will be assessed using Bland-Altman analysis. We will use ICC (two-way mixed-effects, absolute agreement model) to show how closely the measurements resemble each other overall.

Impact of ADL: BI sensor measurements obtained during the standardized ADL protocol will be analyzed to assess susceptibility to motion artifacts. Video recordings will be visually inspected and segmented into movement and no-movement periods. For each segment, the variability of BI measurements (e.g. standard deviation or coefficient of variation) will be compared between movement and no-movement periods. Signal-to-noise ratios and artifact incidence will be quantified to assess real-world feasibility.

Physiological parameters: Concurrent EMG and sitting pressure distribution will be monitored to identify potential interference from muscle activity. Additional stratified analyses will evaluate the influence of autonomic dysregulation on BI accuracy.

### 2.9 Data management and confidentiality

Study data will be recorded using secuTrial (iAS Systems, Berlin, Germany), a web-based data capture and management system with an automated audit trail. Participants will be identified by a unique code. Any identifying information will be stored separately, and access will be restricted. Video recordings will be anonymized through automated blurring. Sensor data will be transmitted via a secure hub and stored on a protected server. All data will be handled in accordance with the Good Clinical Practice (GCP) guideline and national laws to ensure confidentiality and secure storage. Health-related data will be retained for ten years after completion of data collection.

### 2.10 Monitoring and quality assurance

The study will be monitored by GCP-trained personnel who are independent of the study team. A monitoring plan is set-up to ensure that the entered data values are accurate. All entered data will be reviewed and verified by an investigator and signed-off by the principal investigator prior to data export for analyses in statistical software. Specific definitions of data entry fields as well as range and consistency as well as plausibility checks for entered data values will promote data quality. Electronic case report forms will be tested before release. Source data and study documents will be made accessible to monitors and the Ethics Committee for quality assurance purposes. Monitoring reports will be issued after each visit.

### 2.11 Risk-benefit assessment

The study involves minimal risks. Participants may experience anxiety or embarrassment related to recording procedures, and they may be concerned about data protection. These risks are mitigated by voluntary participation, the right to withdraw, and confidentiality. Although participants will not directly benefit from the study, results may contribute to the development of an accurate, noninvasive, wearable bladder volume monitor for individuals with SCI/D and NLUTD. This could optimize catheterization timing and prevent complications associated with bladder overfilling.

## 3 Discussion

This study protocol outlines a multi-phase feasibility trial designed to determine if BI can serve as a noninvasive, wearable method to continuously monitor bladder volume in individuals with SCI/D and NLUTD. The phased approach - progressing from testing in individuals without SCI/D, to validation in individuals with SCI/D, and finally assessment under real-life conditions - provides a comprehensive framework for evaluating technical performance.

Although conventional bladder monitoring techniques, including ultrasound and catheterization, provide valuable clinical information, they are limited by their intermittent nature, reliance on clinical settings, and procedural burden. Ultrasound requires trained operators and correct positioning, making it unsuitable for continuous monitoring. Although portable systems improve mobility, they still require repeated user interaction. Catheterization is the gold standard but is invasive, cannot be applied continuously and associated with risks such as catheter-associated UTIs.

Wearable ultrasound (WUS) systems face additional constraints related to size, power consumption, and accuracy. B-mode systems are precise but require multiple transducers and advanced processing, resulting in bulkier, power-intensive designs. A-mode systems are more compact and more energy-efficient but compromise on imaging resolution and accuracy (24). Commercial WUS devices, such as D-free, SENS-U, and URIKA, provide only qualitative bladder fullness estimates using one- or two-dimensional reconstruction techniques (25). These devices have limited abdominal tissue penetration, which presents a particular challenge in individuals with higher adiposity or who are wheelchair users. Their low-resolution imaging restricts precise real-time volume quantification, and the need for ultrasound gel further limits daily usability. Overcoming these limitations would require major advances in transducer miniaturization, power efficiency, and computational processing.

Other emerging techniques also present limitations. Near-infrared spectroscopy is limited by tissue penetration depth and sensitivity on skin pigmentation, with reported root mean square error and mean absolute error values are 133.7 and 113.4 mL, respectively (26). Electrical impedance tomography (EIT) estimates bladder volume using multi-electrode impedance reconstruction (9) but requires large electrode arrays (n ≥ 8), making it highly sensitive to motion artifacts. Electrode placement is further complicated by the bladder’s anatomical position, particularly when empty and close to the pubic bone, which is especially challenging in pediatric populations and can result in substantial errors (27). High computational demands also limit the feasibility of lightweight wearable EIT systems.

BI technology offers several promising advantages. It enables continuous, dynamic monitoring of bladder filling and emptying. It is noninvasive and unobtrusive, relying on surface electrodes that can be integrated into wearable systems, and it is compatible with daily activities. These features support ambulatory use during transfers, mobility, and other functional tasks, and address unmet clinical needs by enabling individualized catheterization schedules, reducing bladder overdistension, and potentially preventing long-term complications such as recurrent UTIs and renal damage.

Compared with other impedance-based approaches, BI wearables aim to achieve sensitivity to intravesical volume changes using a minimal electrode configuration, providing a technically and ergonomically viable solution. Accordingly, this study focuses on feasibility in terms of usability, accuracy, and reliability under diverse conditions, including variations in sensor design and wearability, electrode placement, signal stability, and confounding factors such as abdominal adiposity, muscle activity, and motion artifacts.

Several challenges remain. The relationship between measured impedance and true bladder volume is influenced not only by urine volume itself, but also by urine concentration, bladder geometry, dynamics of bladder expansion, tissue electrical properties, and electrode-tissue coupling. Urine concentration affects bioimpedance because conductivity depends strongly on ionic concentration. Different urine concentrations due to hydration status, renal output or storage duration may result in different impedance changes for the same volume. This is one reason why Phase 2 includes urodynamic filling with controlled saline concentration. Inter-individual differences in body composition, such as subcutaneous fat thickness, abdominal muscle mass, and visceral fat distribution, can alter current pathways and attenuate bladder-related impedance changes, necessitating subject-specific sensitivity and calibration. Bladder filling is also dynamic and non-uniform, and changes in urine production, bladder shape, posture, and filling rate can modify the effective measurement geometry over time. In addition, there is urine absorption from the bladder (28). These physiological processes will be reflected indirectly by the measured bioimpedance signal, but the small changes in bladder volume may represent a relevant challenge for bioimpedance-based measurements. Thus, sensitivity and limitations of bioimpedance-based measurements under realistic physiological conditions are the central focus of this feasibility study. Furthermore, the lack of pre- and post-voiding volumes during Phases 1 and 3 will not allow to infer absolute bladder volumes with high certainty. However, the aim of this study is not the accurate quantification of bladder volumes, but rather the identification of bladder-related bioimpedance trends, voiding-associated deltas, and the physiological and practical boundary conditions within which this sensing technology may operate reliably. These uncontrolled variations are also representative of realistic ambulatory use scenarios in which a wearable bladder-monitoring system would ultimately be expected to function. Importantly, Phase 2 will provide the controlled reference condition, where bladder filling volume is known during urodynamic testing. Finally, wearable design constraints such as reduced electrode spacing, lower excitation currents, and motion-induced skin–electrode impedance variations, can limit signal-to-noise ratio and measurement stability.

Participants will not receive immediate clinical benefit. However, in the future, users may benefit from improved bladder management and reduced catheter-associated complications through accurate, continuous, and unobtrusive monitoring.

This study is designed to provide essential insights into how these physiological and technical factors affect the accuracy and precision of BI-derived bladder volume estimates, thereby guiding the optimization of sensor design, electrode configurations, and signal-processing algorithms. This exploratory work will serve as a foundation for larger clinical validation studies and eventual clinical implementation. Future studies should address the reliability of BI-derived bladder volume estimates in comparison with ultrasound-derived absolute bladder volumes, which will allow to determine post-void residual urine volumes. If successful, BI wearables could provide a clinically meaningful alternative to current technologies, improve clinical outcomes while reducing healthcare-related complications.
